# Association between circulating biomarkers of endothelial glycocalyx degradation and postoperative acute kidney injury in patients undergoing cardiac surgery with cardiopulmonary bypass

**DOI:** 10.3389/fsurg.2026.1863142

**Published:** 2026-07-13

**Authors:** Minhuan Wang, Min He, Liyun Hong, Yafang Shen, Qin Wang, Yan Sun

**Affiliations:** 1 Department of Anesthesiology, Kunshan First People’s Hospital, Kunshan City, Jiangsu, China; 2 Department of Anesthesiology, Kunshan Women and Children’s Healthcare Hospital, Kunshan City, Jiangsu, China

**Keywords:** acute kidney injury, biomarkers, cardiac surgery, cardiopulmonary bypass, endothelial glycocalyx, heparan sulfate, hyaluronic acid, syndecan-1

## Abstract

**Background:**

Postoperative acute kidney injury remains a frequent and clinically important complication after cardiac surgery with cardiopulmonary bypass, and perioperative biomarkers may help identify patients at increased renal risk.

**Objective:**

To investigate the association between circulating biomarkers of endothelial glycocalyx degradation and postoperative acute kidney injury in patients undergoing cardiac surgery with cardiopulmonary bypass.

**Methods:**

This single-center retrospective observational study included 152 patients who underwent cardiac surgery with cardiopulmonary bypass at Kunshan First People's Hospital. Syndecan-1, hyaluronic acid, and heparan sulfate were measured before cardiopulmonary bypass and at the end of cardiopulmonary bypass. Postoperative acute kidney injury was defined according to the Kidney Disease: Improving Global Outcomes criteria using postoperative serum creatinine and urine output. Between-group comparisons, paired analyses, logistic regression, and receiver operating characteristic analysis were performed.

**Results:**

Postoperative acute kidney injury occurred in 43 of 152 patients, corresponding to an incidence of 28.3%. Patients with acute kidney injury were older, had worse baseline renal function, and underwent longer cardiopulmonary bypass and aortic clamp times. At the end of cardiopulmonary bypass, syndecan-1 and heparan sulfate levels were higher in the acute kidney injury group, whereas hyaluronic acid did not differ significantly between groups. All three glycocalyx biomarkers increased significantly from baseline to the end of cardiopulmonary bypass, with greater increases in the acute kidney injury group, particularly for syndecan-1 and heparan sulfate. In multivariable analysis, older age, longer aortic clamp time, and higher syndecan-1 at the end of cardiopulmonary bypass were associated with postoperative acute kidney injury. Syndecan-1 showed the best discriminatory performance among the evaluated biomarkers, with an area under the curve of 0.743.

**Conclusions:**

Circulating glycocalyx degradation biomarkers increased during cardiac surgery with cardiopulmonary bypass. Among the biomarkers examined, syndecan-1 measured at the end of cardiopulmonary bypass showed the clearest association with postoperative acute kidney injury and the best discriminatory performance.

## 1 Introduction

Acute kidney injury remains one of the most frequent and clinically important postoperative complications after cardiac surgery, particularly in procedures performed with cardiopulmonary bypass (1, 2). According to the literature, even mild postoperative AKI may result in prolonged intensive care stay, longer hospitalization, higher resource use, and worse clinical outcomes (1–3). In clinical practice, the burden of cardiac surgery-associated AKI is amplified by the fact that early renal injury may be incompletely recognized when attention is focused mainly on overt creatinine rise or advanced clinical deterioration (4). For this reason, there has been sustained interest in identifying perioperative markers that may improve risk stratification before established kidney dysfunction becomes fully apparent (2, 3, 5).

The pathogenesis of postoperative AKI is complex and multifactorial. According to the relevant studies, the main reasons could be the advanced age, preexisting renal impairment, diabetes, and prolonged CPB, etc (6). Among these mechanisms, endothelial dysfunction has attracted increasing attention, where endothelial glycocalyx covers the luminal surface of the vascular endothelium and contributes to barrier function, shear stress sensing, regulation of leukocyte-endothelial interaction, and maintenance of microvascular homeostasis (7, 8). According to the related clinical studies, glycocalyx disruption may be associated with inflammatory and ischemic states, which may then therefore contribute to capillary leak, tissue edema, and organ dysfunction (9).

From a clinical perspective, CPB is considered a major contributor to glycocalyx injury, through mechanisms such as nonpulsatile flow, blood contact with artificial surfaces, oxidative stress, and inflammatory activation (10, 11). Earlier perioperative studies demonstrated that glycocalyx shedding can occur during major ischemia-reperfusion procedures in humans (9), and subsequent cardiac surgery studies showed that circulating glycocalyx components, including syndecan-1, heparan sulfate, and hyaluronan, increase during coronary artery bypass procedures and after exposure to CPB (10, 12). Prolonged bypass duration has also been linked to greater syndecan-1 release, further supporting a relationship between procedural burden and endothelial surface injury (11). In parallel, microcirculatory studies in on-pump cardiac surgery have suggested that acute glycocalyx degradation may accompany impaired perfusion and postoperative endothelial dysfunction (12, 13).

Despite these observations, the clinical significance of perioperative glycocalyx shedding in patients undergoing cardiac surgery with CPB has not been fully defined. Existing work has largely focused either on general cardiac surgery populations or on physiological correlates of glycocalyx injury rather than its relation to postoperative renal injury in a relatively homogeneous valve surgery cohort (6, 11). Moreover, although several perioperative risk models for postoperative AKI have been proposed, biomarkers of endothelial glycocalyx degradation have not yet been routinely incorporated into these models (6). Against this background, this research was designed to examine the association between circulating biomarkers of endothelial glycocalyx degradation and postoperative AKI in patients undergoing cardiac surgery with CPB. In this research, syndecan-1, hyaluronic acid, and heparan sulfate were measured before and at the end of CPB. As a result, the perioperative changes, the relationship with postoperative AKI, and the potential discriminatory performance in comparison with conventional perioperative variables were further analyzed and evaluated.

## 2 Materials and methods

### 2.1 Study design and ethics

This was a single-center retrospective observational study conducted at Kunshan First People's Hospital in accordance with the Declaration of Helsinki. The study was approved by the Ethics Committee of Kunshan First People's Hospital (Approval No. 2024-06-027). Written informed consent was obtained from all participants.

### 2.2 Study population

During the study period, 178 patients who underwent cardiac surgery with cardiopulmonary bypass were assessed for eligibility. Patients were eligible for inclusion if complete perioperative measurements of syndecan-1, hyaluronic acid, and heparan sulfate, together with complete postoperative serum creatinine and urine output data required for AKI assessment, were available. Exclusion criteria were age younger than 18 years in 6 patients, preoperative end-stage renal disease or need for dialysis in 7 patients, missing perioperative glycocalyx biomarker measurements in 5 patients, and missing postoperative serum creatinine or urine output data in 8 patients. After these exclusions, 152 patients were included in the final analysis.

### 2.3 Data collection and biomarker measurement

Clinical, operative, and laboratory data were collected from the electronic medical record system, anesthesia records, and the perioperative laboratory database. Baseline and intraoperative variables included age, sex, body mass index, hypertension, diabetes, baseline serum creatinine, baseline estimated glomerular filtration rate, left ventricular ejection fraction, type of surgery, anesthetic regimen, cardiopulmonary bypass duration, aortic clamp time, lactate before and at the end of cardiopulmonary bypass, surgical urgency, and intraoperative blood transfusion. Postoperative variables included serum creatinine at 24, 48, and 72 h, urine output during the first 24 postoperative hours, ICU stay, and total hospital stay.

Peripheral venous blood samples were collected immediately before cardiopulmonary bypass initiation and at CPB completion. Samples were collected into EDTA tubes, centrifuged at 1,000 g for 10 min, and the separated plasma was stored at −80 °C until analysis. Syndecan-1, hyaluronic acid, and heparan sulfate were measured by ELISA using DY2780 (R&D Systems, Minneapolis, MN, USA), CSB-E04805 h, and CSB-E09585 h (Cusabio Biotech Co., Ltd., Wuhan, China). Assay ranges were 125–8,000 pg/mL, 0.156–10 ng/mL, and 20–8,000 ng/mL, respectively. Reported intra-/inter-assay coefficients of variation were <8%/<10%, <8%/<10%, and <10%, respectively. All samples were assayed in duplicate by personnel blinded to postoperative AKI status and other clinical outcomes.

### 2.4 Definition of postoperative AKI

The primary outcome was postoperative AKI. AKI was defined and staged according to the Kidney Disease: Improving Global Outcomes criteria using postoperative serum creatinine and urine output. Patients were categorized into non-AKI and AKI groups for group comparisons. AKI severity was further classified as stage 1, stage 2, or stage 3.

### 2.5 Assessment of perioperative glycocalyx biomarker changes

To characterize perioperative glycocalyx shedding, changes in syndecan-1, hyaluronic acid, and heparan sulfate from before CPB to the end of CPB were evaluated in the overall cohort and separately in the non-AKI and AKI groups. Absolute change was defined as the biomarker level at the end of CPB minus the corresponding preoperative level. These analyses were used to describe perioperative biomarker kinetics and to compare the extent of biomarker increase between patients with and without postoperative AKI.

### 2.6 Statistical analysis

Statistical analyses were performed using IBM SPSS Statistics, version 29.0.1.0 171, IBM Corp., Armonk, NY, USA. Continuous variables are presented as mean ± standard deviation, and categorical variables as number and percentage. Continuous variables were compared using the independent-samples t test, and categorical variables were compared using the chi-square test or Fisher's exact test, as appropriate. Changes in glycocalyx biomarkers from before CPB to the end of CPB were analyzed using paired comparisons. Univariable logistic regression analysis was first performed to identify variables associated with postoperative AKI, followed by multivariable logistic regression analysis. Because baseline serum creatinine and estimated glomerular filtration rate both reflect baseline renal function, estimated glomerular filtration rate was used to represent baseline renal reserve in the multivariable model. Postoperative serum creatinine and urine output were not entered into the regression analyses because they were used in the definition of AKI. Receiver operating characteristic analysis was performed to assess the discriminatory performance of selected biomarkers for postoperative AKI. All tests were two-sided, and a *P*value less than 0.05 was considered statistically significant.

## 3 Results

### 3.1 Patient selection and incidence of postoperative AKI

**Figure 1:**
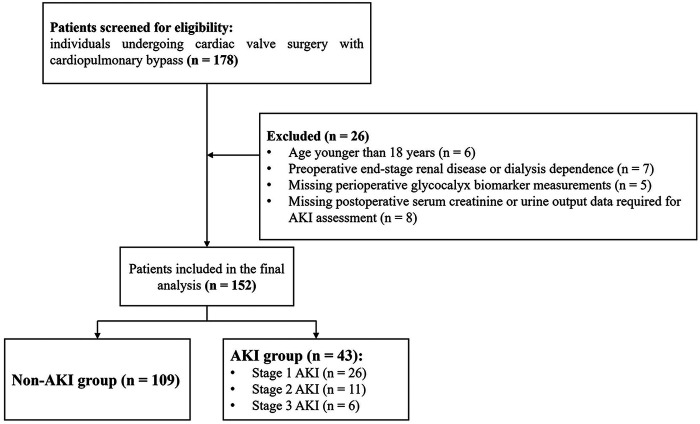
Flowchart of patient selection and postoperative acute kidney injury classification.

A total of 178 patients undergoing cardiac surgery with CPB were screened during the study period. After 26 patients were excluded according to the criteria, 152 patients fulfilled the criteria and were finally analyzed. Among the included population, 43 patients (28.3%) developed postoperative AKI, and 109 patients were categorized as non-AKI (Figure 1). Meanwhile, stage 1 AKI predominated, and lower numbers of patients were assessed as stage 2 and 3 (Table 1).

**Table 1:** Distribution of postoperative AKI stages.

AKI stage	*n* (%)
No AKI	109 (71.7%)
Stage 1	26 (17.1%)
Stage 2	11 (7.2%)
Stage 3	6 (3.9%)

### 3.2 Baseline and intraoperative characteristics

Those who developed postoperative AKI were older and had worse basal renal function with higher basal serum creatinine and lower basal estimated glomerular filtration rate, as compared with non-AKI patients. Diabetes was associated with the AKI group as well. By contrast, sex, body mass index, left ventricular ejection fraction, the proportion of multiple valve surgery, and the use of propofol-based anesthesia did not differ significantly between the two groups (Table 2).

**Table 2:** Baseline and intraoperative characteristics according to postoperative AKI status.

Variable	Overall (*n* = 152)	Non-AKI (*n* = 109)	AKI (*n* = 43)	*P* value
Age, years	62.23 ± 9.29	60.19 ± 9.13	67.40 ± 7.62	<0.001
Male sex, *n* (%)	87 (57.2%)	64 (58.7%)	23 (53.5%)	0.686
BMI, kg/m^2^	24.79 ± 2.86	24.77 ± 2.90	24.85 ± 2.80	0.878
Hypertension, *n* (%)	107 (70.4%)	72 (66.1%)	35 (81.4%)	0.095
Diabetes, *n* (%)	46 (30.3%)	26 (23.9%)	20 (46.5%)	0.011
Baseline SCr, μmol/L	92.49 ± 16.94	89.07 ± 15.82	101.17 ± 16.77	<0.001
Baseline eGFR, mL/min/1.73m^2^	80.30 ± 15.84	84.15 ± 14.86	70.54 ± 14.06	<0.001
LVEF, %	60.36 ± 6.21	60.33 ± 6.18	60.43 ± 6.37	0.927
Multiple valve surgery, *n* (%)	35 (23.0%)	25 (22.9%)	10 (23.3%)	1.000
Propofol-based anesthesia, *n* (%)	63 (41.4%)	46 (42.2%)	17 (39.5%)	0.906
CPB duration, min	127.36 ± 26.60	122.67 ± 25.69	139.25 ± 25.37	<0.001
Aortic clamp time, min	84.84 ± 23.54	80.26 ± 23.30	96.44 ± 20.10	<0.001
Lactate before CPB, mmol/L	1.18 ± 0.27	1.18 ± 0.27	1.18 ± 0.24	0.975
Lactate at end of CPB, mmol/L	2.43 ± 0.66	2.33 ± 0.69	2.68 ± 0.51	<0.001
Emergency surgery, *n* (%)	20 (13.2%)	8 (7.3%)	12 (27.9%)	<0.001
Intraoperative blood transfusion, *n* (%)	54 (35.5%)	30 (27.5%)	24 (55.8%)	0.001

Group differences were also found in terms of the intraoperative characteristics. Patients who developed postoperative AKI had longer CPB duration and longer aortic clamp time. Lactate levels before CPB were comparable between groups, whereas lactate levels at the end of CPB were higher in the AKI group. Emergency surgery and intraoperative blood transfusion were more frequent in patients who developed postoperative AKI.

### 3.3 Perioperative glycocalyx biomarkers and postoperative short-term outcomes

As observed in Table 3, preoperative levels of syndecan-1, hyaluronic acid, and heparan sulfate were generally similar in the two groups, although syndecan-1 tended to be higher in patients who later developed AKI. By the end of CPB, both syndecan-1 and heparan sulfate were higher in the AKI group, while hyaluronic acid did not differ significantly between groups. In addition, the postoperative renal indices differed between groups, where patients with AKI also had longer ICU stay and longer hospital stay than patients without AKI.

**Table 3:** Perioperative glycocalyx biomarkers and postoperative outcomes according to AKI status.

Variable	Overall (*n* = 152)	Non-AKI (*n* = 109)	AKI (*n* = 43)	*P* value
Syndecan-1 before CPB, ng/mL	45.31 ± 10.07	44.36 ± 10.00	47.72 ± 9.92	0.064
Syndecan-1 at end of CPB, ng/mL	86.02 ± 18.24	81.77 ± 17.50	96.78 ± 16.03	<0.001
Hyaluronic acid before CPB, ng/mL	54.01 ± 11.06	53.41 ± 10.73	55.54 ± 11.78	0.307
Hyaluronic acid at end of CPB, ng/mL	79.59 ± 17.17	78.27 ± 16.40	82.92 ± 18.82	0.160
Heparan sulfate before CPB, ng/mL	15.70 ± 2.95	15.74 ± 3.02	15.61 ± 2.81	0.801
Heparan sulfate at end of CPB, ng/mL	22.01 ± 5.18	21.11 ± 5.15	24.28 ± 4.53	<0.001
Postoperative SCr at 24 h, μmol/L	98.98 ± 22.39	92.52 ± 18.41	115.33 ± 22.33	<0.001
Postoperative SCr at 48 h, μmol/L	102.89 ± 24.67	93.63 ± 18.35	126.37 ± 23.33	<0.001
Postoperative SCr at 72 h, μmol/L	102.04 ± 25.68	93.50 ± 20.81	123.66 ± 26.44	<0.001
Urine output in first 24 h, mL	1,809.41 ± 328.76	1,879.87 ± 318.16	1,630.93 ± 255.83	<0.001
ICU stay, days	3.10 ± 1.24	2.71 ± 1.09	4.10 ± 1.09	<0.001
Hospital stay, days	13.31 ± 3.14	12.43 ± 2.79	15.54 ± 2.79	<0.001

### 3.4 Perioperative changes in circulating glycocalyx degradation biomarkers

**Figure 2:**
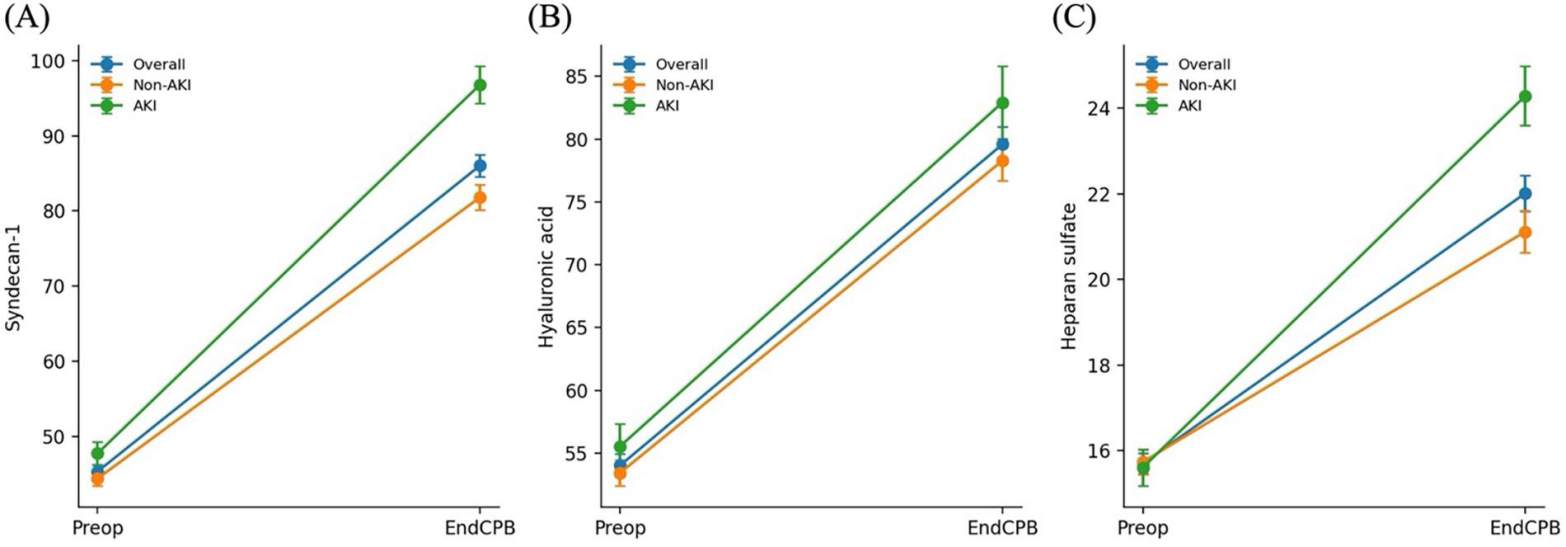
Perioperative changes in circulating endothelial glycocalyx degradation biomarkers in the overall cohort and according to postoperative AKI status. **(A)** Perioperative changes in circulating syndecan-1 levels. **(B)** Perioperative changes in circulating hyaluronic acid levels. **(C)** Perioperative changes in circulating heparan sulfate levels.

Syndecan-1, hyaluronic acid, and heparan sulfate all increased significantly from before CPB to the end of CPB in the overall cohort, and a similar within-group pattern was seen in both the non-AKI and AKI groups (Table 4, Figure 2). The increase was more pronounced in patients with AKI, especially for syndecan-1 and heparan sulfate. Although hyaluronic acid changed in the same direction, the between-group difference was less apparent.

**Table 4:** Perioperative changes in glycocalyx biomarkers.

**Group**	**Biomarker**	**Preop, mean ± SD**	**EndCPB, mean ± SD**	***Δ*, mean ± SD**	***P* value**
**Overall**	Syndecan-1	45.31 ± 10.07	86.02 ± 18.24	40.71 ± 15.70	<0.001
	Hyaluronic acid	54.01 ± 11.06	79.59 ± 17.17	25.57 ± 12.41	<0.001
	Heparan sulfate	15.70 ± 2.95	22.01 ± 5.18	6.31 ± 4.25	<0.001
**Non-AKI**	Syndecan-1	44.36 ± 10.00	81.77 ± 17.50	37.41 ± 14.94	<0.001
	Hyaluronic acid	53.41 ± 10.73	78.27 ± 16.40	24.86 ± 12.01	<0.001
	Heparan sulfate	15.74 ± 3.02	21.11 ± 5.15	5.37 ± 4.20	<0.001
**AKI**	Syndecan-1	47.72 ± 9.92	96.78 ± 16.03	49.06 ± 14.57	<0.001
	Hyaluronic acid	55.54 ± 11.78	82.92 ± 18.82	27.38 ± 13.33	<0.001
	Heparan sulfate	15.61 ± 2.81	24.28 ± 4.53	8.68 ± 3.42	<0.001

### 3.5 Univariable logistic regression analysis

As summarized in Table 5, several baseline, intraoperative, and biomarker-related variables were associated with postoperative AKI in the univariable logistic regression analysis. Among baseline characteristics, older age, diabetes, higher serum creatinine, and lower estimated glomerular filtration rate were associated with a greater likelihood of AKI. Intraoperatively, longer CPB duration, longer aortic clamp time, higher lactate levels at the end of CPB, emergency surgery, and intraoperative blood transfusion were also associated with the outcome. With respect to glycocalyx-related biomarkers, higher syndecan-1 and heparan sulfate levels at the end of CPB were associated with postoperative AKI, while preoperative syndecan-1 and hypertension showed borderline associations. By contrast, no significant associations were identified for sex, body mass index, left ventricular ejection fraction, multiple valve surgery, propofol-based anesthesia, lactate before CPB, hyaluronic acid, or preoperative heparan sulfate.

**Table 5:** Univariable logistic regression analysis for postoperative AKI.

Variable	Effect	OR (95% CI)	*P* value
Age	Per year	1.10 (1.05–1.15)	<0.001
Male sex	Male vs. female	0.81 (0.40–1.65)	0.558
BMI	Per kg/m^2^	1.01 (0.89–1.14)	0.878
Hypertension	Yes vs. no	2.25 (0.95–5.34)	0.066
Diabetes	Yes vs. no	2.78 (1.32–5.84)	0.007
Baseline SCr	Per μmol/L	1.05 (1.03–1.08)	<0.001
Baseline eGFR	Per mL/min/1.73m^2^	0.94 (0.91–0.97)	<0.001
LVEF	Per %	1.00 (0.95–1.05)	0.927
Multiple valve surgery	Multiple vs. single	1.02 (0.44–2.34)	0.97
Propofol-based anesthesia	Propofol vs. sevoflurane	0.89 (0.44–1.81)	0.742
CPB duration	Per min	1.03 (1.02–1.05)	<0.001
Aortic clamp time	Per min	1.03 (1.02–1.05)	<0.001
Lactate before CPB	Per mmol/L	0.99 (0.24–4.10)	0.994
Lactate at end of CPB	Per mmol/L	2.28 (1.35–3.84)	0.002
Syndecan-1 before CPB	Per ng/mL	1.03 (1.00–1.07)	0.07
Syndecan-1 at end of CPB	Per ng/mL	1.05 (1.03–1.08)	<0.001
Hyaluronic acid before CPB	Per ng/mL	1.02 (0.99–1.05)	0.311
Hyaluronic acid at end of CPB	Per ng/mL	1.02 (1.00–1.05)	0.164
Heparan sulfate before CPB	Per ng/mL	0.99 (0.88–1.11)	0.801
Heparan sulfate at end of CPB	Per ng/mL	1.13 (1.04–1.23)	0.004
Emergency surgery	Emergency vs. elective	4.89 (1.83–13.03)	0.002
Intraoperative blood transfusion	Yes vs. no	3.33 (1.60–6.93)	0.001

### 3.6 Multivariable logistic regression analysis

After adjustment for the selected covariates, older age, longer aortic clamp time, and higher Syndecan-1 at the end of CPB remained associated with postoperative AKI, whereas emergency surgery and intraoperative blood transfusion were not independently associated with the outcome (Table 6).

**Table 6:** Multivariable logistic regression analysis for postoperative AKI.

Variable	OR (95% CI)	*P* value
Age	1.11 (1.03–1.19)	0.005
Hypertension	2.45 (0.75–8.02)	0.138
Diabetes	2.22 (0.76–6.47)	0.144
Baseline eGFR	0.96 (0.93–1.00)	0.063
CPB duration	1.02 (0.99–1.05)	0.196
Aortic clamp time	1.04 (1.00–1.08)	0.028
Lactate at end of CPB	1.94 (0.76–4.97)	0.167
Emergency surgery	1.50 (0.28–7.99)	0.631
Intraoperative blood transfusion	0.35 (0.06–1.96)	0.233
Syndecan-1 at end of CPB	1.04 (1.01–1.07)	0.014

### 3.7 Receiver operating characteristic analysis

Receiver operating characteristic analysis showed that syndecan-1 at the end of CPB had the best discriminatory performance among the evaluated biomarkers, with an area under the curve of 0.743. Nevertheless, heparan sulfate and lactate measured at the end of CPB showed moderate discrimination, whereas hyaluronic acid had limited discriminatory value (Table 7).

**Table 7:** ROC analysis of selected biomarkers for postoperative AKI.

Variable	AUC
Syndecan-1 at end of CPB	0.743
Heparan sulfate at end of CPB	0.667
Lactate at end of CPB	0.668
Hyaluronic acid at end of CPB	0.555

## 4 Discussion

Among these patients who had cardiac surgery with CPB, postoperative AKI was present in 28.3%, and stage 1 accounted for the majority (14). After the final model, older age, poorer baseline renal function, longer aortic clamp time, and greater end-of-bypass syndecan-1 remained associated with postoperative AKI (15). These trends are broadly consistent with the current concept of cardiac surgery-associated AKI, whereby postoperative renal injury is the result of both patient-related vulnerability and bypass-related factors (16). Recent reviews continue to report CPB as a milieu favoring the occurrence of renal hypoperfusion, hemodilution, inflammation, and endothelial dysfunction (15), and studies directed at bypass management have also associated longer clamp or bypass duration with increased AKI risk.

In addition, the perioperative increase of syndecan-1, hyaluronic acid, and heparan sulfate in this study is in line with other studies assessing glycocalyx damage in cardiac surgery (17). Endothelial glycocalyx-focused reviews have elaborated that CPB is an environment that may encourage shedding of the glycocalyx layers by inflammatory activation, ischemia-reperfusion, modified shear stress, and contact with artificial surfaces (18). More recent perioperative studies have similarly reported an increase in circulating shedding markers during on-pump cardiac surgery, with the magnitude of association between specific markers and postoperative outcomes differing among cohorts (19). Bol et al. employed multimodal analyses and proposed that none of the markers can completely represent glycocalyx injury, while Passov et al. found that syndecan-1 and heparan sulfate demonstrated dynamic fluctuations in the early reperfusion phase following cardiac surgery (17).

Among the biomarkers examined here, syndecan-1 appeared to provide the clearest signal. It was higher at the end of CPB in patients who developed AKI, remained associated with AKI after adjustment, and showed the highest AUC among the tested markers. This pattern resembles several recent reports in cardiac surgery. Miyazaki et al. examined perioperative serum syndecan-1 in patients undergoing cardiac surgery with CPB and reported an association with postoperative AKI (20). Kim et al. found that higher preoperative syndecan-1 was related to severe AKI after adult cardiac surgery with CPB (21), and Ay et al. reported a similar signal in patients undergoing isolated coronary artery bypass grafting with CPB (22). More recent literature has also linked elevated postoperative syndecan-1 to AKI progression or kidney replacement therapy, and adult cardiac surgery data have suggested that syndecan-1 may be related not only to AKI occurrence but also to progressive AKI in the setting of fluid overload (13, 23).

Heparan sulfate showed a weaker but still detectable association, whereas hyaluronic acid was less informative in the between-group comparisons and ROC analysis. This uneven performance across markers is not unusual in the glycocalyx literature. Prior studies of bypass-related glycocalyx injury have reported that different shedding products may change in parallel over time yet differ in their association with clinical endpoints, suggesting that they reflect overlapping but not identical aspects of endothelial disturbance (17). The pediatric bypass literature has also shown substantial perioperative glycocalyx shedding, but the relationship between marker kinetics and organ injury has not been uniform across analytes or time points (19, 24). Against that background, the present data are consistent with perioperative glycocalyx injury, while also indicating that syndecan-1 was the most informative of the biomarkers assessed here.

The conventional perioperative variables in this study remained relevant. Older age and lower baseline renal reserve were associated with postoperative AKI, which is consistent with prior work on cardiac surgery-associated kidney injury. Aortic clamp time remained associated with AKI after adjustment, whereas CPB duration did not, possibly because these two variables partly overlap in what they reflect about procedural complexity and ischemic exposure. Lactate at the end of CPB was associated with AKI in univariable analysis but not after adjustment. This result is not entirely out of line with earlier reports. Several studies have linked higher perioperative lactate levels to postoperative AKI after cardiac surgery, but the independent role of lactate has not been consistent across models and sampling strategies. Some datasets have identified lactate as an independent predictor, whereas others have suggested that it functions more as a marker of perioperative stress severity than as a stand-alone determinant (25–27).

These results should be interpreted with several limitations in mind. The study was observational and single-center in design, so the associations identified here should not be read as causal. The sample size was moderate, which may have limited precision for some estimates, particularly for covariates that showed only borderline significance. Biomarkers were measured at selected perioperative time points rather than serially across the full postoperative period, and circulating markers were used as indirect indicators rather than direct structural measurements of the glycocalyx. At the same time, the study also has practical strengths. It focused on a relatively homogeneous surgical population, incorporated several glycocalyx-related biomarkers, and evaluated the data using complementary approaches that included between-group comparison, temporal change analysis, logistic regression, and ROC analysis. Within these limits, the findings suggest that perioperative glycocalyx shedding, especially syndecan-1 release, may be relevant to postoperative renal risk assessment in cardiac surgery with CPB. Because blood samples were obtained in the setting of CPB, circulating biomarker concentrations may have been influenced by hemodilution. Formal normalization or adjustment for dilutional effects was not performed in the present study, and this should be considered when interpreting the absolute biomarker levels.

## 5 Conclusion

In patients undergoing cardiac surgery with CPB, circulating glycocalyx degradation biomarkers increased during the perioperative period. Among the biomarkers examined, syndecan-1 measured at the end of CPB showed the most consistent association with postoperative AKI and the best discrimination among the tested biomarkers. These findings support further evaluation of glycocalyx-related biomarkers as adjuncts to perioperative renal risk assessment, particularly in combination with conventional clinical variables.

## Data Availability

The original contributions presented in the study are included in the article/Supplementary Material, further inquiries can be directed to the corresponding author.
